# Marketing techniques, health, and nutritional claims on processed foods and beverages before and after the implementation of mandatory front-of-package warning labels in Peru

**DOI:** 10.3389/fnut.2022.1004106

**Published:** 2022-11-02

**Authors:** Lorena Saavedra-Garcia, Ximena Taboada-Ramirez, Akram Hernández-Vásquez, Francisco Diez-Canseco

**Affiliations:** CRONICAS Center of Excellence in Chronic Diseases, Universidad Peruana Cayetano Heredia, Lima, Peru

**Keywords:** food marketing, front-of-package warning labels, marketing techniques, health claims, nutritional claims, Peru

## Abstract

In June 2019, mandatory front-of-package warning labels (FOPL) were implemented in Peru. The aim of the study was to describe changes in marketing strategies on packaging: marketing techniques (MT), health claims (HC), and nutritional claims (NC) on the packaging of products frequently consumed by children before and after the FOPL implementation. Product photos were taken pre- (March 2019) and post-implementation (March-October 2020) in three supermarkets in Lima, Peru. Following INFORMAS protocols and Peruvian Technical Norms, the presence of MT, HC, and NC was assessed on all package sides. Products were classified as “high-in” and “not high-in” based on the regulation threshold for critical nutrients. Differences in the proportion of products with each strategy in both periods were evaluated. Also, a subsample of products was matched according to the barcode and exact McNemar test was used to compare proportions of strategies pre/post-implementation. We included 883 and 1,035 products in pre- and post-implementation, respectively. In both periods, MT appeared on almost 70% of all products. The presence of HC increased significantly only for beverages (24.5–38.1%, *p* < 0.001). In both phases, NC were commonly used on beverages (>80%). Overall, the prevalence of “high-in” products using MT increased (73.6–82.1%, *p* = 0.007), while use of HC increased among “not high-in” products (32.9–41.6%, *p* < 0.001). There is a high frequency of MT on all products and NC on beverages. The increase in MT in “high-in” products may be an industry response to minimize the impact of the FOPL on food choices and sales. New regulatory aspects regarding labeling should be implemented to strengthen the current policy.

## Introduction

Worldwide, traditional dietary patterns are being replaced by unhealthy patterns, characterized by the consumption of ultra-processed food, driven by food marketing strategies, and other factors (1). Marketing and other strategies used by the food industry have been effective in making ultra-processed food sales grow exponentially and consequently, increasing their consumption. This rise has been associated with an increase in obesity, higher waist circumference, lower levels of high-density lipoprotein (HDL) cholesterol, and more negative health effects such as metabolic, cardiovascular, and cerebrovascular diseases (2, 3). According to the Pan American Health Organization, Peru has the highest growth in per capita sales of ultra-processed food in Latin America, increasing from 179 kcal per capita/day in 2009 to 207 kcal per capita/day in 2014 (4).

Food and beverage packaging is commonly used for marketing purposes. Marketing techniques such as cartoons, games, and gifts (i.e., toys) (5) as well as claims (affirmations regarding properties or benefits about the product or ingredients) are often used to influence consumers’ food choices (6). Children are especially vulnerable to such techniques because of their inability to distinguish marketing from other content (7), and susceptibility to marketing and peer perceptions, which influences their food perception and choices (8). For instance, children tend to perceive products that include cartoons as more fun than those without cartoons and to like those with cartoons better (5, 9). Moreover, children show higher taste preferences for food packaging with the presence of characters, granting this marketing technique a positive influence over their purchase intentions (10). On the other hand, adults are more influenced by information cues on the package (8), and the evidence shows that parents tend to assess the healthfulness of a product based on nutritional claims (11). Unfortunately, marketing strategies in food and beverage packaging are more frequently used for promoting energy-dense and nutrient-poor foods than nutrient-dense ones (12); for instance, cartoons are more prevalent on products with a less healthy nutritional profile (13–15). In addition, the presence of health and nutritional claims can create a “health halo” effect when consumers generalize the benefit claimed to the overall healthfulness of the product (16), like fruit drinks that claim to have no artificial sweeteners or to be 100% natural, but contain high amounts of sugar, which often misleads parents to choose products that appear to be healthy for their children, when objectively they are not (17).

In response to the potentially negative impact of food industry marketing on the population’s diets and health, some countries have linked food and beverage marketing regulations to mandatory “front-of-package warning labels” (FOPL) requirements for products “high-in” nutrients of concern, such as sugar, saturated fat, and sodium. In 2012, Chile banned child-directed prices and advertising attractive to children under 14 years old on products “high-in” energy and nutrients of concern (18). After this policy’s implementation, a reduction in child-directed advertising strategies was seen on cereal products, decreasing from 36 to 21% (19). More recently, Mexico (20) and Argentina (21) passed similar policies, banning the use of cartoons and games in products that have FOPL. Additionally, Argentina restricted the use of health and nutritional claims on products carrying a FOPL (21).

In 2013, the Peruvian Government passed the “Law of Promotion of Healthy Eating for Children and Adolescents” (Law 30021) (22). According to the Law, products that exceed specific thresholds for nutrients of concern such as sugar, saturated fat, or sodium, or contain *trans*-fat, must carry a black octagon-shaped FOPL with the phrase “high-in” (sugar/saturated fat/sodium) or “contains *trans*-fats”, accompanied by the message “avoid excessive consumption” or “avoid its consumption”, respectively, on the packaging and all types of advertisements about them (e.g., tv, social media, radio). The thresholds established by the law are being implemented in two phases; the first phase became effective in June 2019 and the second phase in September 2021 (Supplementary Table 1; 23). Although the law includes some regulations focused on food advertising (e.g., prohibiting claims about improving physical strength or popularity, or suggesting parents will be more intelligent if they purchase the product), it does not extend to banning marketing techniques and health and nutritional claims on packaging.

The introduction of mandatory FOPL in the Peruvian market requires many processed products to carry them, which may potentially decrease sales of some foods and beverages, and could potentially motivate the food industry to use different marketing strategies to promote their products. Given that Peruvian Law does not ban marketing techniques, or health, and nutritional claims, we hypothesized that the Peruvian food industry may change the type or frequency of marketing strategies on food and beverage packaging, especially on products that will carry a FOPL, in order to maintain their sales. This study aims to identify and describe the changes in marketing techniques, health, and nutritional claims in the packaging of food and non-alcoholic beverages after the implementation of the FOPL in Peru, and to evaluate those differences according to the nutritional quality of the products (“high-in” or “not high-in”). Findings will may inform advocates and policymakers about the strategies frequently used by industry that could negatively affect the achievement of the FOPL policy objective.

## Materials and methods

### Study design and setting

We compared data from two cross-sectional collections (before and after the implementation of the FOPL policy) from processed products sold in supermarkets in Lima, the capital city of Peru. Additionally, a subsample of products was matched according to the barcode that allowed a pre/post-implementation analysis. For the pre-implementation period, we collected pictures of all the available packaged foods and non-alcoholic beverages in three supermarkets, between March and April 2019, 3 months before the implementation of the FOPL policy. The three supermarkets had a nationwide presence and each one targeted different socioeconomic levels of the population (high, medium, and low). In the post-implementation period, our team went back to the same stores between March and October 2020 to carry out the post-implementation data collection.

### Outcome variables

The outcomes of interest in this study were the pre- and post-implementation differences in the proportion of products using the following marketing strategies on packaging: (i) nine marketing techniques, based on the INFORMAS (International Network for Food and Obesity / Non-communicable Diseases Research, Monitoring and Action Support) protocol (24), and (ii) four health, and (iii) four nutritional claims adapted from the Peruvian Technical Norms for food claims (25), a document that establishes features from products and services. These 17 variables, their definitions, and examples of each can be found in Table 1. Of the 17 marketing strategies, only gifts are prohibited in advertisements of products directed to children less than 16 years old (22).

**TABLE 1:** Variables: Marketing strategies assessed.

Marketing strategy on packaging	Description	Examples
**Marketing techniques**		
Characters	Images, photographs, drawings, and caricatures of real or fictional characters	Cartoons, athletes, celebrities, images, or photos from boys and girls < 18 years
Sports	Any character playing sports, an invitation for sports events or event sponsorship, presence of any object that refers to a sport	Children playing sports, a logo indicating the brand sponsors a team
Donations	Products whose packaging shows messages from public welfare organizations	The brand’s charitable foundation
Price	Promotions referring to the price	Messages such as “get more for less,” an additional percentage of the product, discount coupons, and low price
Gifts	Packaging includes the free delivery of an object with the purchase of the product	Chocolate eggs with a surprise, toys, or stickers inside the package
Contests	Packaging announces that the consumer can access a contest, redemption, raffle, or similar competitions through an additional action after the product purchase	Scan the QR code on the box to participate or subscribe to a contest for a trip
Logos	The packaging contains a food system labeling or an endorsement logo of a certain scientific society	GDA logo, the logo with the approval of the Dietician’s Society
Lifestyles	Motivational phrases, advice, and tips in the packaging to lead a healthy lifestyle	Messages promoting a healthy lifestyle such as “It is good to exercise”
Marketing directed to children	Packaging intended to appeal to children	Games, playful products, products shapes, messages related to fun, fonts, or graphics allusive to fun, special lines such as “mini”
**Health claims**		
Nutrient message and function	Messages that describe and focus on the physiological role of a nutrient in growth, development, normal functions, or biological activities of the body, and not on disease reduction. The message must include the nutrient plus the specific function	“Mixture of malt with vitamins and minerals that help the release of energy, muscular function, and maintenance of bones,” or “With probiotics and fiber that help you regulate your intestinal transit”
Disease risk reduction message	Messages that emphasize the relationship between a specific food consumption (without specifying a particular nutrient or ingredient) and reducing the risk of developing a disease	“Can Help Lower Cholesterol as part of a Heart Healthy Diet”
General health message	Messages associated with specific food consumption (without specifying a particular nutrient or ingredient) with general health benefits	“Brings you energy”
Fantasy terms	Invented words that refer to the product’s nutritional content	“Calcifem” (product enriched with calcium oriented to women)
**Nutritional claims**		
Ingredient related message	The packaging shows messages indicating that the product contains healthy ingredients or messages that indicate the product does not contain unhealthy ingredients	“With Andean grains” and “0% artificial colors”
Nutritional content	The packaging mentions a nutrient, mentions the amount of a nutrient, mentions the energy value, or mentions that the product has no specific nutrient	“With omega 3 and 9,” “0% *trans*-fat,” “High in dietetic fiber,” “It is a protein source,” and “Provides Calcium and Phosphorus”
Nutritional comparison	Messages that compare the nutrient level or energetic value of two or more foods with terms that indicate one product has more or less of a nutrient	“95% reduced in fat, compared to a whole yogurt”
Non-caloric sweetener addition	Messages that indicate the product has the addition of non-caloric sweeteners, apart from the ingredient list	“Partially sweetened with Stevia”

Moreover, the status of products according to the FOPL policy (“high-in” and “not high-in”) was assessed based on the parameters of Peruvian Law No. 30021 (Supplementary Table 1).

### Food and beverages sample

Photographs of all sides of each product sold in the supermarkets were taken by trained nutritionists. After each round of data collection, quality control for every photo was conducted to verify that the text and images were clear. If not, new photographs were taken. Then, the label data from the pictures were recorded in the REDCap database hosted at the University of North Carolina at Chapel Hill (26). Product name, brand, weight, container, and nutritional composition per portion or per 100 g or 100 mL were entered in the database.

For this study, we selected the eight categories of foods and beverages most consumed by children and adolescents (5, 27, 28; Supplementary Table 2):

(a) Beverages: (i) nectars, (ii) flavored drinks (*“refrescos”*), (iii) carbonated drinks, and (iv) dairy drinks.

(b) Foods: (i) bakery products, (ii) breakfast cereals, (iii) desserts, and (iv) snacks.

The selected categories included data from a total of 1,153 processed products collected before the implementation (in 2019) and 1,238 after the implementation (in 2020). If a product had two or more packaging types, only the smallest one was selected, considering that these are frequently directed to children and offered at school cafeterias and kiosks. As a result, 270 and 203 products were excluded in 2019 and 2020, respectively. Finally, we evaluate the use of marketing techniques and health and nutritional claims on 883 products in 2019 and 1,035 in 2020.

To determine the status of products according to the FOPL policy, it was necessary to evaluate the nutritional composition. Products without a nutrition facts panel were excluded and multipacks with more than one nutrition facts panel were excluded (179 in 2019 and 203 in 2020). Also, products requiring reconstitution were excluded from this analysis (126 in 2019 and 102 in 2020) because many packages did not provide preparation instructions or exact amounts of added ingredients, or the weight or portion size of the prepared product were not available.

**FIGURE 1:**
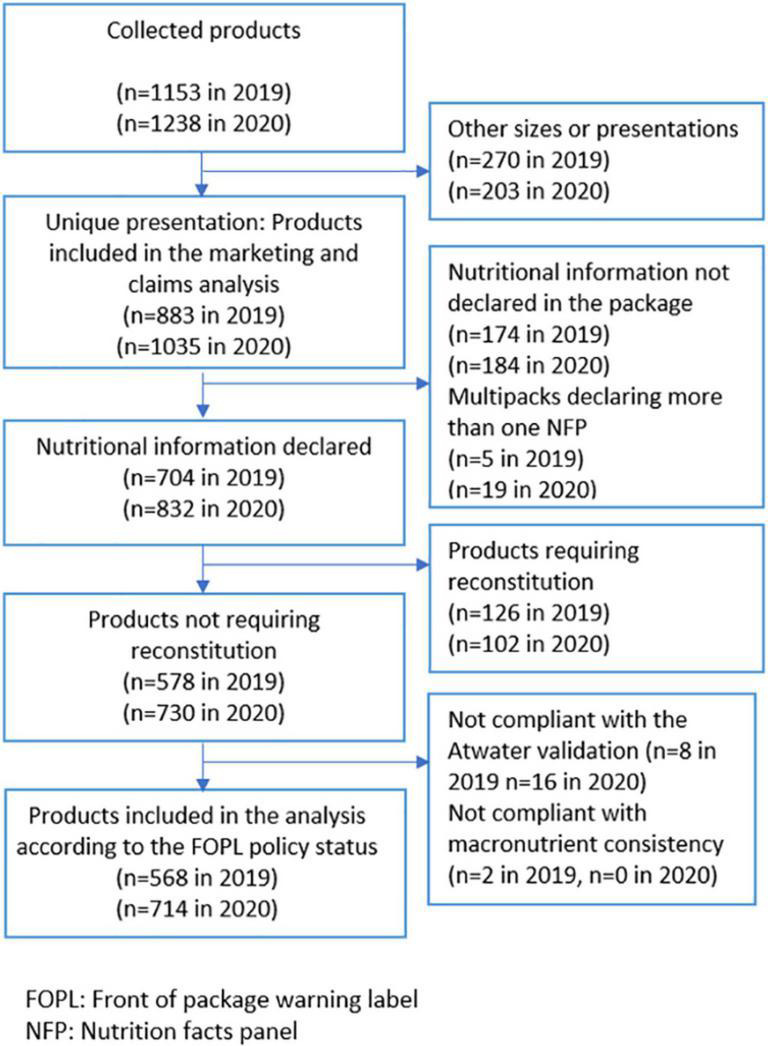
Sample flowchart.

To identify inconsistencies, the Atwater System calculation was used to check the total energy declared in the label with the sum of energy provided by each macronutrient using Atwater’s constants (i.e., fats = 9 kcal/g, proteins = 4 kcal/g, carbohydrates = 4 kcal/g). This validation was applied to all products that declared each of the three macronutrients and energy. Foods with total energy values that equaled or were within 20% of the Atwater calculation for energy were included (29). Eight products from the 2019 collection and 16 from 2020 did not comply and were excluded from the nutritional composition analysis. Total sugar was compared to total carbohydrates for each product that provided both values. Products with total sugar greater than total carbohydrates were omitted from the analysis (two in 2019 and no products in 2020). Likewise, products with an amount of saturated fat that exceeded the amount of total fat were also reviewed, but no product was excluded from the analysis (Figure 1).

Dairy drinks were excluded from the analysis of *trans*-fat content because a determination could not be made as to whether the *trans*-fat amount declared in the nutritional information table was added or intrinsic in dairy products.

### Coding of marketing techniques and claims

The methodology aimed to identify the presence of different marketing techniques, health claims, and nutritional claims on each product. First, two trained Peruvian nutritionists coded a random sample of 20% of products from the pre- and post-implementation period. The nutritionists coded the absence or presence of each type of marketing technique or claim, and where it was located (front or side/back). The presence of a marketing technique or claim was recorded only once, even if it was repeated multiple times on the same product. The percentage of agreement for each of the 31 variables was calculated. The agreement ranged from 93 to 100%. The lowest agreement was for nutrition claims related to nutritional messages. The discrepancies were reviewed and resolved by the two nutritionists. In the second stage, one nutritionist coded all remaining products.

#### Categorization of products according to the law

To determine if a product was categorized as “high-in” or “not high-in,” the information declared in the nutrition facts panel was compared to the thresholds established for the first phase of implementation of the FOPL policy, in June 2019 (Supplementary Table 1). Products from the pre- and post-implementation period were considered “high-in” if they exceeded thresholds for any of the nutrients of concern: sugar, saturated fat, or sodium, or contained *trans*-fat, thus receiving at least one octagon. All products with nutrients below the thresholds were categorized as “not high-in”.

### Data analysis

The frequency of each marketing technique and claim was calculated overall and by food and beverage category. Chi-squared and Fisher’s exact tests were used to evaluate differences in proportions between the pre- and post-implementation periods. The proportion of marketing techniques and claims in each period was compared according to the product’s regulation categorization (“high-in” or “not high-in”) based on the nutrient thresholds for the first phase of Peru’s FOPL regulation. For the subsample of matched products, we used exact McNemar test to compared proportions pre- and post-implementation in the outcomes of interest.

Analysis was conducted using the statistical software package Stata v15 (STATA Corp, College Station TX, USA). A *p*-value of less than 0.05 was deemed statistically significant.

### Ethics

This project was approved by the Institutional Ethical Committee at Universidad Peruana Cayetano Heredia, Lima, Peru (project 102750). Additionally, the supermarkets granted permission to collect information.

## Results

For the analysis of the marketing techniques and health and nutritional claims in the present study, a total of 883 products in the pre-implementation phase (2019) and 1,035 in post-implementation (2020) were included for the cross sectional analysis. Almost one-third of the products in each phase were beverages, 31.0% (*n* = 274) and 32.5% (*n* = 336) in the pre- and post-implementation phases, respectively. The category with the most products in the beverage group was “Nectars” in the pre- and post-implementation (28.5%, *n* = 78, and 38.0%, *n* = 104), while among foods, “Bakery products” predominated in both collections (48.9%, *n* = 298 and 57%, *n* = 350; Table 2). A total of 321 products were collected in both phases and considered for the longitudinal analysis, 29.6% (*n* = 95) were beverages and 70.4% (*n* = 226) were foods.

**TABLE 2:** Marketing techniques, health, and nutritional claims before and after front-of-package warning labels policy implementation, cross sectional analysis.

Category	Total products		Any marketing technique			Any health claim			Any nutrition claim		
	Pre-implementation *n* (%)	Post-implementation *n* (%)	Pre-implementation *n* (%)	Post-implementation *n* (%)	*P*-value	Pre-implementation *n* (%)	Post-implementation *n* (%)	*P*-value	Pre-implementation *n* (%)	Post-implementation *n* (%)	*P*-value
**Beverages**	274 (100.0)	336 (100.0)	182 (66.4)	217 (64.6)	0.635	67 (24.5)	128 (38.1)	**<0.001**	227 (82.9)	282 (83.9)	0.721
Nectars	78 (28.5)	104 (31.0)	31 (39.7)	53 (51.0)	0.133	14 (18.0)	40 (38.5)	**0.003**	65 (83.3)	87 (83.7)	0.954
Flavored drinks	69 (25.2)	58 (17.3)	54 (78.3)	45 (77.6)	0.927	11 (15.9)	11 (19.0)	0.654	64 (92.8)	56 (96.6)	0.350
Carbonated drinks	57 (20.8)	83 (24.7)	36 (63.2)	53 (63.9)	0.933	7 (12.3)	9 (10.8)	0.793	40 (70.2)	56 (67.5)	0.735
Dairy drinks	70 (25.5)	91 (27.0)	61 (87.1)	66 (72.5)	0.024	35 (50.0)	68 (74.7)	**0.001**	58 (82.9)	83 (91.2)	0.111
**Foods**	609 (100.0)	699 (100.0)	427 (70.1)	507 (72.5)	0.335	101 (16.6)	130 (18.6)	0.341	255 (41.9)	308 (44.1)	0.425
Bakery products	298 (48.9)	350 (50.1)	175 (58.7)	252 (72.0)	**<0.001**	18 (6.0)	31 (8.9)	0.176	84 (28.2)	103 (29.4)	0.728
Breakfast cereals	117 (19.2)	144 (20.6)	101 (86.3)	121 (84.0)	0.605	77 (65.8)	84 (58.3)	0.216	106 (90.6)	123 (85.4)	0.204
Desserts	58 (9.5)	61 (8.7)	54 (93.1)	49 (80.3)	**0.041**	2 (3.5)	5 (8.2)	0.440	22 (37.9)	29 (47.5)	0.29
Snacks	136 (22.3)	144 (20.6)	97 (71.3)	85 (59.0)	**0.031**	4 (2.9)	10 (6.9)	0.171	43 (31.6)	53 (36.8)	0.361

Comparisons of proportions of marketing techniques, health, and nutritional claims in products from pre- *vs.* post-implementation period were made using Chi-squared and Fisher’s exact tests.

Bold values represent *p* < 0.05.

**FIGURE 2:**
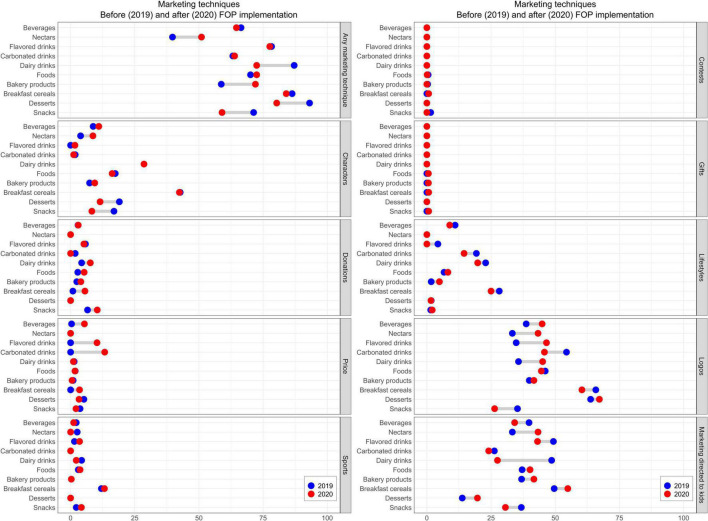
Marketing techniques used before and after the front-of-package warning label policy implementation, cross sectional analysis.

In both periods, almost seven out of ten products displayed at least one of the ten marketing techniques, with an average of 1.3 (range 0–5) and 1.4 (range 0–5) in the pre- and post-implementation phase, respectively. However, the prevalence was higher in some categories, such as “Desserts” in the pre-implementation period and “Breakfast cereals” in the post-implementation, in which almost nine out of ten products displayed a marketing technique (Table 2). The findings presented in Figure 2 show that in both phases, the most used marketing technique was “Logos” (most products carried a GDA logo), followed by “Marketing directed to children”, which were both used on around 40% of the products (Figure 2). The least commonly used techniques were “Gifts” and “Contests” (Supplementary Table 3). Three categories (“Dairy drinks,” “Desserts,” and “Snacks”) significatively reduced the prevalence of marketing techniques after the FOPL implementation (*p* < 0.05). Notably, the reduction of marketing techniques on “Dairy drinks” was due to the lower number of products carrying a marketing technique directed to children (48.6–27.5%, *p* = 0.006). On the other hand, some categories increased the use of marketing techniques, with a significant increase on “Bakery products” (*p* < 0.001; Table 2), the same trend was observed in the longitudinal analysis in this food category (*p* < 0.001; Supplementary Table 4).

**FIGURE 3:**
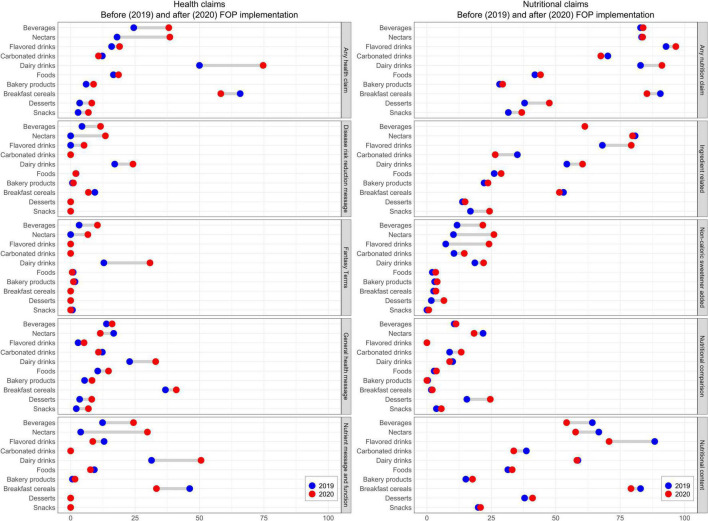
Health and nutritional claims used before and after the front-of-package warning labels policy implementation, cross sectional analysis.

In regard to claims, health claims were used less frequently than nutritional claims, with a mean of 0.3 (range 0–3) and 0.5 (range 0–3) health claims per product in the pre- and post-implementation phase, respectively. Two categories had a higher proportion of products with health claims in comparison to other categories: “Dairy drinks” and “Breakfast cereals,” in which 50 to almost 75% of products had health claims in both phases. Few products in “Carbonated drinks” and “Desserts” displayed health claims (Table 2). As shown in Figure 3, in both phases, the most used health claims were “General health message” and “Nutrient message and function”, while fewer products used “Fantasy terms”. Changes in the prevalence of any health claims were observed among beverages, where they increased by almost 15% (24.5–38.1%, *p* < 0.001) between phases, in contrast to the cross-sectional analysis, the longitudinal analysis showed increases in the use of this type of claim only in “Nectars” (*p* = 0.016; Supplementary Table 4). Only one food category, “Breakfast cereals” had a reduction in the use of one type of health claim (“Nutrient message and function”) (46.2–33.3%, *p* = 0.035; Supplementary Table 5).

In contrast to health claims, nutritional claims were frequently used, with a mean of 1.0 claim (range 0–4) per product in both periods. These claims were especially common on beverages, where almost four out of five products in each period presented any nutritional claim. In both phases, the category with the most claims was “Flavored drinks”, while “Bakery products” was the category with fewest claims of this type (Table 3). In addition, the most used nutritional claims were “Nutritional content” and “Ingredient related” (Figure 3). It is worth noting that a large number of pre-implementation period products already used nutrition claims (particularly ingredient-related claims). Although no changes were observed in the proportion of products carrying a nutritional claim, when examining changes by specific claim, “non-caloric sweetener added” (NCS) claims increased notably among beverages, raising from 11.7 to 21.7% (*p* = 0.001) in the post-implementation phase (Supplementary Table 6).

**TABLE 3:** Differences in the percentage of products using marketing techniques, health, and nutritional claims according to the front of package warning labels policy status, cross sectional analysis.

	*n* (2019)	Pre-implementation (2019)	*n* (2020)	Post-implementation (2020)	Difference (%)	*P*-value
**(1) Marketing techniques**						
% of “not high-in” products with at least one marketing technique	216	77.8	363	72.2	–5.6	0.136
Beverages	109	82.6	192	63.0	–19.6	**<0.001**
Foods	107	72.9	171	82.5	9.6	0.058
% of “high-in” products with at least one marketing technique	352	73.6	351	82.1	8.5	**0.007**
Beverages	81	55.6	61	68.9	13.3	0.107
Foods	271	79.0	290	84.8	5.8	0.071
% of total products with at least one marketing technique	568	75.0	714	77.0	2.0	0.407
Beverages	190	71.1	253	64.4	–6.7	0.141
Foods	378	77.3	461	84.0	6.7	**0.014**
**(2) Health claims**						
% of “not high-in” products with at least one health claim	216	32.9	363	41.6	8.7	**0.037**
Beverages	109	34.9	192	43.2	8.3	0.155
Foods	107	30.8	171	39.8	9	0.132
% of “high-in” products with at least one health claim	352	18.2	351	18.2	0	0.986
Beverages	81	18.5	61	47.5	29	**<0.001**
Foods	271	18.1	290	12.1	–6.0	**0.046**
% of total products with at least one health claim	568	23.8	714	30.1	6.3	**0.011**
Beverages	190	27.9	253	44.3	16.4	**<0.001**
Food	378	21.7	461	22.3	0.6	0.806
**(3) Nutritional claims**						
% of “not high-in” products with at least one nutritional claim	216	79.2	363	81.3	2.1	0.537
Beverages	109	87.2	192	91.7	4.5	0.209
Foods	107	71.0	171	69.6	–1.4	0.799
% of “high-in” products with at least one nutritional claim	352	52.8	351	47.6	–5.2	0.163
Beverages	81	87.7	61	78.7	–9.0	0.151
Foods	271	42.4	290	41.0	–1.4	0.737
% of total products with at least one nutritional claim	568	62.9	714	64.7	1.8	0.492
Beverages	190	87.4	253	88.5	1.1	0.707
Foods	378	50.5	461	51.6	1.1	0.752

“High-in” products are products exceeding at least one parameter in sugar, saturated fats or containing *trans*-fat according to the first phase of the Peruvian law.

Comparisons of proportions of marketing techniques and claims in products from pre- *vs*. post-implementation period were made using Chi-squared and Fisher’s exact tests. Bold values represent *p* < 0.05.

In the analyses based on FOPL policy status, the use of marketing techniques on foods and beverages classified as “high-in” increased by almost 10% (73.6–82.1%, *p* = 0.007). Among “not high-in” products, the use of these strategies on beverages decreased by almost 20% (82.6–63.0%, *p*-value < 0.001); in contrast, marketing strategies on foods increased almost 10% (72.9–82.5%, *p* = 0.058). The prevalence of health claims on “not high-in” foods and beverages increased by 8.7% (32.9–41.6%, *p* = 0.037) and on “high in” beverages by 29% (18.5–47.5%, *p* < 0.001). No statistically significant changes in the use of nutritional claims were observed when products were categorized according to FOPL policy status (Table 3). In the longitudinal analysis we confirmed that products “high-in” in the pre-implementation phase (2019) increase the use of marketing techniques after the implementation (*p* = 0.007), in addition “high-in” products increase the use of health claims (*p* = 0.012; Supplementary Table 7).

## Discussion

The present study analyzed a wide range of marketing strategies on food and beverage packaging, including marketing techniques, and health, and nutritional claims. Findings suggest an extended use of marketing techniques and nutritional claims, and to a lesser extent, of health claims, before and after the implementation of the FOPL policy in Peru. After the implementation, some changes, including increases and decreases, were observed in the use of marketing techniques and claims among the studied food and beverage categories. Of particular concern, the use of marketing techniques increased among “high-in” products.

Of the three types of marketing strategies on packaging analyzed, marketing techniques were more commonly used in all food and beverage categories; in contrast, claims were more prevalent in some specific categories. This is an expected result given that marketing techniques include a large variety of strategies that can be used on any product, ranging from the use of logos to specific colors on the packaging. In contrast, health, and nutritional claims require that the product contains specific ingredients or components that confer certain properties and benefits (6). Additionally, it is important to note the high percentage of products–especially beverages- using nutritional claims even before the FOPL policy implementation (30), and at the same time the lower use of health claims. Our results from the pre-implementation phase are similar to those found in Mexico. In both countries, nutritional claims were used more than health claims (57 *vs.* 25% in Peru, and 33.8 *vs.* 3.4% in Mexico, respectively), with “Nutritional content” claims being the most common nutritional claims (31).

Among the different marketing techniques assessed, the “Logos” and “Marketing directed to children” were the most used in both periods. Most of the “Logos” used on the products analyzed in this study were from the GDA system, a front-of-package label system (32) frequently promoted by the food industry (33). It could be interest to study in further studies if the presence of both systems (GDA and FOPL octagons) interact and influence food choices. Another marketing technique used frequently in our sample is “Marketing directed to children” (around 40% overall products in both phases). The prevalence of the use of this technique was similar in Chile (36%) before the implementation of the Chilean FOPL policy in 2016 (19). This similarity may be due to geographic context (both are South American countries) and shared food suppliers. It has been reported that “marketing directed to children” techniques are frequently used on products “high-in” nutrients of concern, promoting the selection of those products (17). Similarly, a previous study in Peru warned about the frequent use of marketing directed to children on products high in sugar (14).

In both phases, two product categories that frequently use marketing strategies, especially marketing techniques and health claims, were “Dairy drinks” and “Breakfast cereals”. This aligns with a study in Costa Rica (13), where “Breakfast cereals” had the highest use of promotional marketing strategies. Traditionally, these products have been considered healthy, especially for children, because they often use marketing techniques and claims that create a “health halo” effect that makes parents believe that a product is healthy and based their food selection on that impression (11). Interestingly, these two categories experienced changes in the use of marketing strategies after the implementation of the FOPL policy. For instance, in the post-implementation period, the proportion of “Dairy drinks” using marketing techniques decreased, but the percentage using health and nutritional claims increased. One possible explanation is that the industry increased the use of claims to reinforce the idea that their products are healthy, and to counter customer concerns about the nutritional composition of packaged foods during the months close to the implementation of the FOPL policy.

Even though we found no changes in the use of nutritional claims overall, when we analyzed each type of claim separately, we observed a significant increase in the use of messages related to NCS. This could reflect a greater use of NCS instead of added sugar in beverages, in order to avoid the “high-in sugar” octagon. Importantly, in some contexts, such as Mexico (34), the use of NCS could be perceived as positive because it signifies a reduction of (or no increase in) calories (35), and in some cases, the addition of a natural NCS could be perceived as healthier due to its natural origin (36). However, considering their possible adverse health effects (35), countries such as Mexico, are implementing warning messages for products using NCS (20).

After the implementation of the FOPL in Peru, the use of some strategies rose among “high-in” products. Cross-sectional and longitudinal analysis shown that marketing techniques increased overall products, but also, we observed a large increase in the use of health claims among “high-in” beverages in the cross sectional analysis and overall products in the longitudinal one. The increased use of those strategies on “high-in” products could be interpreted as a food industry response to minimize the impact of the octagons. Moreover, in the cross-sectional analysis the “not high-in” products were using more health claims to highlight the “healthy” properties of their products, as they are not carrying an octagon and could represent a healthier alternative. This could also be explained by the growth and development of the health food market nowadays, due to consumers’ growing interest in healthy lifestyles and wellness (37).

### Strengths and limitations

Our study included a large sample of processed products from different food and beverage categories. Additionally, our evaluation included all package sides, since the whole package can include marketing strategies; in contrast, previous studies were limited to the front of packages (13, 14).

The study also has some limitations. First, the set of products included were those available in the three supermarkets. Products from small retailers and other points of sales such as kiosks and *bodegas* were not collected. However, the stores visited are nationwide supermarkets targeting different socioeconomic groups. Additionally, only some categories of food and beverages were included, and we limited our sample to one type of packaging per product. In that sense, our results are not representative of all the products offered in the Peruvian market. Furthermore, our results on the subsample of matched products are limited by the small sample size and the power to detect statistical differences. However, since the Peruvian law is focused on children and adolescents, it is more important to analyze changes in products that are usually consumed by children and adolescents. Also, this study did not include information regarding sales data from the analyzed products. Further studies would be needed to explore if changes in marketing strategies are related to product sales. On the other hand, it is also possible that the marketing strategies coded by only one nutritionist could introduce personal bias, however, we anticipated this by training and coding a proportion of products by two fieldworkers to standardize the criteria used to classify.

Finally, the second data collection was carried out during the COVID-19 pandemic and was extended for 8 months due to the lockdown and social restrictions. Thus, some products from 2020 differ from those of 2019, especially seasonal products (e.g., baked products, ice creams, Easter chocolate eggs), and imported products that had limited availability in the Peruvian market during the lockdown.

### Impact on public health

There have been some relevant changes to the marketing strategies on packages used by the food industry after the implementation of the FOPL policy in Peru. The increased use of marketing techniques among products carrying the “high-in” warning label is especially relevant for the aim of the Law of Promotion of Healthy Eating. Notably, NCS claims rose significantly, providing evidence of the increased use of these ingredients. Currently, the Peruvian Law restricts some advertising strategies of products directed to children less than 16 years; however, of all the strategies assessed in this study, only gifts are restricted by the law.

To boost the effects of the Peruvian law, policymakers could ban the use of some marketing techniques and claims assessed in this research on “high-in” products and add warning messages for NCS to avoid misunderstandings regarding the nutritional value of products and better inform consumers. Other Latin American countries such as Chile, Mexico, and Argentina have implemented these policies. In Chile, after restricting child-directed marketing for “high-in” products, a study found a decrease in the proportion of “high-in” breakfast cereals that used child-directed strategies and an increase in the “not high-in” products that used child-directed strategies (*p* < 0.005 for both cases) (19).

Finally, even though the study found significant changes in some food categories and marketing strategies after the implementation of FOPL policy, the results show that most “high-in” products already used either marketing techniques or claims, and that it is increasing. So, it is important to inform and raise public awareness about how to evaluate the nutritional quality of a product, not only based on the content of nutrients of concern, which may be insufficient for some populations to identify healthy products (28), but also based on different traits such as ingredients, processing, and labeling. In Peru, the current public policies like the Law of Promotion of Healthy Eating (22), the Healthy Eating Guidelines (38) and the National Multisectoral Health Policy to 2030 (39) allow the implementation of other actions such as implementing communication-based or educational interventions for the clear interpretation of product healthfulness and informed decision-making regarding healthy food purchases.

## Conclusion

This study found a high use of marketing on beverages and food packaging, especially for marketing techniques and nutritional claims, before and after the implementation of the FOPL policy in Peru. Some decreases in the use of marketing techniques were observed among specific food categories, but an increase in health claims was observed for beverages. Additionally, the use of marketing techniques on “high-in” products increased, while the prevalence of health claims increased on “not high-in” products after the implementation.

To support the aim of promoting healthy eating among children and adolescents, we recommend developing communication and educational campaigns to inform the public about food labeling features such as FOPL, and nutritional facts panel. Additionally, new regulatory measures to limit the use of marketing techniques, health, and nutritional claims on “high-in” products should be implemented to strengthen the current FOPL policy.

## Data availability statement

The raw data supporting the conclusions of this article will be made available by the authors, without undue reservation.

## Author contributions

LS-G and XT-R: conceptualization, methodology, and writing – original draft preparation. AH-V: data curation and formal analysis. LS-G: supervision. FD-C, LS-G, and AH-V: resources and funding acquisition. FD-C: project administration. All authors contributed to revising the manuscript for important content, edit, read, and agreed to the published version of the manuscript.

## Abbreviations

FOPL: front-of-package warning labels
MT: marketing techniques
HC: health claims
NC: nutritional claims
COVID-19: coronavirus disease 2019.
