# Supplementary material for: Marketing techniques, health, and nutritional claims on processed foods and beverages before and after the implementation of mandatory front-of-package warning labels in Peru
Source: Front Nutr. 2022 Nov 2;9:1004106. doi: 10.3389/fnut.2022.1004106 (PMC9666783; doi:10.3389/fnut.2022.1004106)
Supplement: Supplementary file 1 [file Data_Sheet_1.docx]

| **Table S1. Technical parameters and entry into force of the “Law of Promotion of Healthy Eating for Children and Adolescents”** | | | |
| --- | --- | --- | --- |
| **Nutrient of concern** | **Product** | **Term of Entry into Force** | |
|  |  | **After 6 Months of Approval of the Advertising Warning Manual** | **After 39 Months of Approval of the Advertising Warning Manual** |
| Total Sugars | Solids | Greater or equal to 22.5 g/100 g | Greater or equal to 10 g/100 g |
|  | Beverages | Greater or equal to 6 g/100 mL | Greater or equal to 5 g/100 mL |
| Saturated Fats | Solids | Greater or equal to 6 g/100 g | Greater or equal to 4 g/100 g |
|  | Beverages | Greater or equal to 3 g/100 mL | Greater or equal to 3 g/100 mL |
| Sodium | Solids | Greater or equal to 800 mg/100 g | Greater or equal to 400 mg/100 mL |
|  | Beverages | Greater or equal to 100 mg/100 mL | Greater or equal to 100 mg/100 mL |
| Trans fats | Solids and Beverages | Contains trans fats | Not allowed the addition of trans fats |

|  |
| --- |
|  |

**Table S2. Description of beverages and products included in this study**

| **Category** | **Description** | **Examples** |  |
| --- | --- | --- | --- |
|  |  |  |  |
| ***Beverages*** | | |  |
| *Nectars* | Artificial juices with pulp | nectars, artificial juices |  |
| *Flavored drinks* | Soft beverages flavored | apple water, *chicha* (boiled corn drink), |  |
| *Carbonated drinks* | Carbonated soft beverages | Sodas with or no sugar added, flavored carbonated drinks |  |
| *Dairy drinks* | Beverages with milk or milk based | drinkable yogurt, chocolate milk |  |
| ***Foods*** | | |  |
| *Bakery products* | Cereal or grain based baked products | cookies, crackers, sweet pastries, muffins |  |
| *Breakfast cereals* | Ceral or grain based | cereals, granola, oat, muesli |  |
| *Desserts* | Non cereal based desserts | jelly, pudin, freeze desserts |  |
| *Snacks* | Salty snacks | potato chips, corn based snacks, pretzels |  |

| **Table S3. Marketing techniques before (2019) and after (2020) FOPL policy implementation, cross-sectional analysis (continues)** | | | | | | | | | | | | | | | | |  |
| --- | --- | --- | --- | --- | --- | --- | --- | --- | --- | --- | --- | --- | --- | --- | --- | --- | --- |
|  |  |  |  |  |  |  |  |  |  |  |  |  |  |  |  |  |  |
| **Category** | **Total Before (n)** | **Total After (n)** | **Any marketing technique** | | | **Characters** | | | **Sports** | | | **Donations** | | | **Price** | | |
|  |  |  | Before n(%) | After n(%) | p value | Before n(%) | After n(%) | p value | Before n(%) | After n(%) | p value | Before n(%) | After n(%) | p value | Before n(%) | After n(%) | p value |
| **Beverages** | 274 | 336 | 182 (66.4) | 217 (64.6) | 0.635 | 24 (8.8) | 37 (11.0) | 0.356 | 6 (2.2) | 4 (1.2) | 0.357 | 8 (2.9) | 10 (3.0) | 0.967 | 1 (0.4) | 18 (5.4) | **<0.001** |
| *Nectars* | 78 | 104 | 31 (39.7) | 53 (51.0) | 0.133 | 3 (3.9) | 9 (8.7) | 0.239 | 2 (2.6) | 0 (0.0) | 0.182 | - | - | - | - | - | **-** |
| *Flavored drinks* | 69 | 58 | 54 (78.3) | 45 (77.6) | 0.927 | 0 (0.0) | 1 (1.7) | 0.457 | 1 (1.5) | 2 (3.5) | 0.592 | 4 (5.8) | 3 (5.2) | 1.000 | 0 (0.0) | 6 (10.3) | **0.008** |
| *Carbonated drinks* | 57 | 83 | 36 (63.2) | 53 (63.9) | 0.933 | 1 (1.8) | 1 (1.2) | 1.000 | - | - | - | 1 (1.8) | 0 (0.0) | 0.407 | 0 (0.0) | 11 (13.3) | **0.003** |
| *Dairy drinks* | 70 | 91 | 61 (87.1) | 66 (72.5) | **0.024** | 20 (28.6) | 26 (28.6) | 1.000 | 3 (4.3) | 2 (2.2) | 0.653 | 3 (4.3) | 7 (7.7) | 0.516 | 1 (1.4) | 1 (1.1) | 1.000 |
| **Foods** | 609 | 699 | 427 (70.1) | 507 (72.5) | 0.335 | 106 (17.4) | 113 (16.2) | 0.549 | 18 (3.0) | 26 (3.7) | 0.445 | 17 (2.8) | 37 (5.3) | **0.023** | 11 (1.8) | 12 (1.7) | 0.902 |
| *Bakery products* | 298 | 350 | 175 (58.7) | 252 (72.0) | **<0.001** | 22 (7.4) | 33 (9.4) | 0.352 | 1 (0.3) | 1 (0.3) | 1.000 | 7 (2.4) | 14 (4.0) | 0.237 | 3 (1.0) | 2 (0.6) | 0.666 |
| *Breakfast cereals* | 117 | 144 | 101 (86.3) | 121 (84.0) | 0.605 | 50 (42.7) | 61 (42.4) | 0.952 | 14 (12.0) | 19 (13.2) | 0.766 | 1 (0.9) | 8 (5.6) | **0.045** | 0 (0.0) | 5 (3.5) | 0.067 |
| *Desserts* | 58 | 61 | 54 (93.1) | 49 (80.3) | **0.041** | 11 (19.0) | 7 (11.5) | 0.254 | - | - | - | - | - | - | 3 (5.2) | 2 (3.3) | 0.674 |
| *Snacks* | 136 | 144 | 97 (71.3) | 85 (59.0) | 0.031 | 23 (16.9) | 12 (8.3) | 0.030 | 3 (2.2) | 6 (4.2) | 0.502 | 9 (6.6) | 15 (10.4) | 0.256 | 5 (3.7) | 3 (2.1) | 0.491 |

Comparisons of proportions of marketing techniques, health and nutritional claims in products from pre- versus post-implementation period were made using Chi-squared and Fisher's exact tests. Bold values represent p<0.05.

| **Table S3. Marketing techniques before (2019) and after (2020) FOPL policy implementation, cross-sectional analysis (continuation)** | | | | | | | | | | | | | | | | | |
| --- | --- | --- | --- | --- | --- | --- | --- | --- | --- | --- | --- | --- | --- | --- | --- | --- | --- |
|  |  |  |  |  |  |  |  |  |  |  |  |  |  |  |  |  |  |
| **Category** | **Total Before (n)** | **Total After (n)** | **Gifts** | | | **Contests** | | | **Logos** | | | **Lifestyles** | | | **Marketing directed to children** | | |
|  |  |  | Before n(%) | After n(%) | p value | Before n(%) | After n(%) | p value | Before n(%) | After n(%) | p value | Before n(%) | After n(%) | p value | Before n(%) | After n(%) | p value |
| Beverages | 274 | 336 | - | - | - | - | - | - | 106 (38.7) | 151 (44.9) | 0.120 | 30 (11.0) | 30 (8.9) | 0.405 | 109 (39.8) | 115 (34.2) | 0.157 |
| *Nectars* | 78 | 104 | - | - | - | - | - | - | 26 (33.3) | 45 (43.3) | 0.174 | - | - | - | 26 (33.3) | 45 (43.3) | 0.174 |
| *Flavored drinks* | 69 | 58 | - | - | - | - | - | - | 24 (34.8) | 27 (46.6) | 0.178 | 3 (4.3) | 0 (0.0) | 0.250 | 34 (49.3) | 25 (43.1) | 0.487 |
| *Carbonated drinks* | 57 | 83 | - | - | - | - | - | - | 31 (54.4) | 38 (45.8) | 0.317 | 11 (19.3) | 12 (14.5) | 0.448 | 15 (26.3) | 20 (24.1) | 0.766 |
| *Dairy drinks* | 70 | 91 | - | - | - | - | - | - | 25 (35.7) | 41 (45.1) | 0.232 | 16 (22.9) | 18 (19.8) | 0.635 | 34 (48.6) | 25 (27.5) | 0.006 |
| Foods | 609 | 699 | 0 (0.0) | 4 (0.6) | 0.128 | 3 (0.5) | 1 (0.1) | 0.253 | 281 (46.1) | 312 (44.6) | 0.585 | 41 (6.7) | 57 (8.2) | 0.330 | 226 (37.1) | 281 (40.2) | 0.253 |
| *Bakery products* | 298 | 350 | 0 (0.0) | 2 (0.6) | 0.502 | 1 (0.3) | 0 (0.0) | 0.460 | 119 (39.9) | 146 (41.7) | 0.646 | 5 (1.7) | 17 (4.9) | **0.026** | 110 (36.9) | 146 (41.7) | 0.213 |
| *Breakfast cereals* | 117 | 144 | 0 (0.0) | 1 (0.7) | 1.000 | 0 (0.0) | 1 (0.7) | 1.000 | 77 (65.8) | 87 (60.4) | 0.370 | 33 (28.2) | 36 (25.0) | 0.559 | 58 (49.6) | 79 (54.9) | 0.395 |
| *Desserts* | 58 | 61 | - | - | - | - | - | - | 37 (63.8) | 41 (67.2) | 0.695 | 1 (1.7) | 1 (1.6) | 1.000 | 8 (13.8) | 12 (19.7) | 0.391 |
| *Snacks* | 136 | 144 | 0 (0.0) | 1 (0.7) | 1.000 | 2 (1.5) | 0 (0.0) | 0.235 | 48 (35.3) | 38 (26.4) | 0.106 | 2 (1.5) | 3 (2.1) | 1.000 | 50 (36.8) | 44 (30.6) | 0.271 |

Comparisons of proportions of marketing techniques, health and nutritional claims in products from pre- versus post-implementation period were made using Chi-squared and Fisher's exact tests. Bold values represent p<0.05.

**Table S4. Marketing techniques, health and nutritional claims before and after front-of-package warning labels policy implementation, longitudinal subsample**

| **Category** | **Post**  **Pre** | | | **Any marketing technique** | | | | | **Any Health Claim** | | | | | | **Any Nutrition Claim** | | | | | | | |  |  |  |  |
| --- | --- | --- | --- | --- | --- | --- | --- | --- | --- | --- | --- | --- | --- | --- | --- | --- | --- | --- | --- | --- | --- | --- | --- | --- | --- | --- |
|  |  |  |  | **Yes (n)** | **No (n)** | | | **p value** | **Yes (n)** | | | **No (n)** | | **p value** | **Yes (n)** | | | | **No (n)** | | | **p value** | | |  |  |
| **Beverages (n = 95)** | | Yes (n) | 62 | | | 1 | NS | | | 28 | 1 | | NS | | | 82 | | 0 | | | NS | | | |  | |
|  | | No (n) | 0 | | | 32 |  | | | 7 | 59 | |  | | | 1 | | 12 | | |  | | | |  | |
| Nectars (n = 29) | | Yes (n) | 15 | | | 0 | NS | | | 7 | 0 | | **0.016** | | | 27 | | 0 | | | NS | | | |  | |
|  | | No (n) | 0 | | | 14 |  | | | 7 | 15 | |  | | | 0 | | 2 | | |  | | | |  | |
| Flavored drinks (n = 15) | | Yes (n) | 11 | | | 0 | NS | | | 3 | 0 | | NS | | | 15 | | 0 | | | NS | | | |  | |
|  | | No (n) | 0 | | | 4 |  | | | 0 | 12 | |  | | | 0 | | 0 | | |  | | | |  | |
| Carbonated drink (n = 27) | | Yes (n) | 17 | | | 0 | NS | | | 4 | 1 | | NS | | | 20 | | 0 | | | NS | | | |  | |
|  | | No (n) | 0 | | | 10 |  | | | 0 | 22 | |  | | | 1 | | 6 | | |  | | | |  | |
| Dairy drinks (n = 24) | | Yes (n) | 19 | | | 1 | NS | | | 14 | 0 | | NS | | | 20 | | 0 | | | NS | | | |  | |
|  | | No (n) | 0 | | | 4 |  | | | 0 | 10 | |  | | | 0 | | 4 | | |  | | | |  | |
| **Foods (n = 226)** | | Yes (n) | 172 | | | 6 | **0.009** | | | 43 | 1 | | NS | | | 104 | | 7 | | | NS | | | |  | |
|  | | No (n) | 20 | | | 28 |  | | | 7 | 175 | |  | | | 7 | | 108 | | |  | | | |  | |
| Bakery products (n = 122) | | Yes (n) | 82 | | | 0 | **<0.001** | | | 7 | 1 | | NS | | | 26 | | 3 | | | NS | | | |  | |
|  | | No (n) | 16 | | | 24 |  | | | 0 | 114 | |  | | | 6 | | 87 | | |  | | | |  | |
| Breakfast cereals (n = 57) | | Yes (n) | 56 | | | 1 | NS | | | 35 | 0 | | NS | | | 55 | | 1 | | | NS | | | |  | |
|  | | No (n) | 0 | | | 0 |  | | | 2 | 20 | |  | | | 0 | | 1 | | |  | | | |  | |
| Desserts (n = 5) | | Yes (n) | 5 | | | 0 | NS | | | 0 | 0 | | NS | | | 5 | | 0 | | | NS | | | |  | |
|  | | No (n) | 0 | | | 0 |  | | | 3 | 2 | |  | | | 0 | | 0 | | |  | | | |  | |
| Snacks (n = 42) | | Yes (n) | 29 | | | 5 | NS | | | 1 | 0 | | NS | | | 18 | | 3 | | | NS | | | |  | |
|  | | No (n) | 4 | | | 4 |  | | | 2 | 39 | |  | | | | 1 | 20 | |  | | | |  | |  |

Comparisons of proportions of marketing techniques, health and nutritional claims in products from pre- versus post-implementation period were made using McNemar exact tests.

NS: not significant

**Table S5. Health claims before (2019) and after (2020) FOPL policy implementation, cross-sectional analysis**

|  |  |  |  |  |  |  |  |  |  |  |  |  |  |  |  |  |  |
| --- | --- | --- | --- | --- | --- | --- | --- | --- | --- | --- | --- | --- | --- | --- | --- | --- | --- |
| **Category** | **Total Before (n)** | **Total After (n)** | **Any Health Claim** | | | **Nutrient message and function** | | | **Disease risk reduction message** | | | **General health message** | | | **Fantasy Terms** | | |
|  |  |  | **Before n(%)** | **After n(%)** | **p value** | **Before n(%)** | **After n(%)** | **p value** | **Before n(%)** | **After n(%)** | **p value** | **Before n(%)** | **After n(%)** | **p value** | **Before n(%)** | **After n(%)** | **p value** |
| Beverages | 274 | 336 | 67 (24.5) | 128 (38.1) | **<0.001** | 34 (12.4) | 82 (24.4) | **<0.001** | 12 (4.4) | 39 (11.6) | **0.001** | 38 (13.9) | 54 (16.1) | 0.450 | 9 (3.3) | 35 (10.4) | **0.001** |
| *Nectars* | 78 | 104 | 14 (18.0) | 40 (38.5) | **0.003** | 3 (3.9) | 31 (29.8) | **<0.001** | 0 (0.0) | 14 (13.5) | **<0.001** | 13 (16.7) | 12 (11.5) | 0.320 | 0 (0.0) | 7 (6.7) | **0.020** |
| *Flavored drinks* | 69 | 58 | 11 (15.9) | 11 (19.0) | 0.654 | 9 (13.0) | 5 (8.6) | 0.428 | 0 (0.0) | 3 (5.2) | 0.093 | 2 (2.9) | 3 (5.2) | 0.659 | - | - | **-** |
| *Carbonated drinks* | 57 | 83 | 7 (12.3) | 9 (10.8) | 0.793 | - | - | - | - | - | - | 7 (12.3) | 9 (10.8) | 0.793 | - | - | **-** |
| *Dairy drinks* | 70 | 91 | 35 (50.0) | 68 (74.7) | **0.001** | 22 (31.4) | 46 (50.6) | **0.015** | 12 (17.1) | 22 (24.2) | 0.278 | 16 (22.9) | 30 (33.0) | 0.159 | 9 (12.9) | 28 (30.8) | **0.007** |
| Foods | 609 | 699 | 101 (16.6) | 130 (18.6) | 0.341 | 56 (9.2) | 54 (7.7) | 0.339 | 13 (2.1) | 14 (2.0) | 0.867 | 64 (10.5) | 103 (14.7) | **0.022** | 6 (1.0) | 4 (0.6) | 0.528 |
| *Bakery products* | 298 | 350 | 18 (6.0) | 31 (8.9) | 0.176 | 2 (0.7) | 6 (1.7) | 0.299 | 2 (0.7) | 4 (1.1) | 0.692 | 16 (5.4) | 29 (8.3) | 0.146 | 5 (1.7) | 4 (1.1) | 0.739 |
| *Breakfast cereals* | 117 | 144 | 77 (65.8) | 84 (58.3) | 0.216 | 54 (46.2) | 48 (33.3) | 0.035 | 11 (9.4) | 10 (6.9) | 0.468 | 43 (36.8) | 59 (41.0) | 0.487 | - | - | - |
| *Desserts* | 58 | 61 | 2 (3.5) | 5 (8.2) | 0.440 | - | - | - | - | - | - | 2 (3.5) | 5 (8.2) | 0.440 | - | - | - |
| *Snacks* | 136 | 144 | 4 (2.9) | 10 (6.9) | 0.171 | - | - | - | - | - | - | 3 (2.2) | 10 (6.9) | 0.087 | 1 (0.7) | 0 (0.0) | 0.486 |

Comparisons of proportions of marketing techniques, health and nutritional claims in products from pre- versus post-implementation period were made using Chi-squared and Fisher's exact tests. Bold values represent p<0.05.

| **Table S6. Nutritional claims before (2019) and after (2020) FOPL policy implementation, cross-sectional analysis** | | | | | | | | | | | | | | | | | |  |
| --- | --- | --- | --- | --- | --- | --- | --- | --- | --- | --- | --- | --- | --- | --- | --- | --- | --- | --- |
|  |  |  |  |  |  |  |  |  |  |  |  |  |  |  |  |  |  | |
| **Category** | **Total Before (n)** | **Total After (n)** | **Any Nutrition Claim** | | | **Ingredient related** | | | **Nutritional content** | | | **Nutritional comparison** | | | **Non-caloric sweetener added** | | | |
|  |  |  | **Before n(%)** | **After n(%)** | **p value** | **Before n(%)** | **After n(%)** | **p value** | **Before n(%)** | **After n(%)** | **p value** | **Before n(%)** | **After n(%)** | **p value** | **Before n(%)** | **After n(%)** | **p value** | |
| Beverages | 274 | 336 | 227 (82.9) | 282 (83.9) | 0.721 | 168 (61.3) | 206 (61.3) | 0.999 | 176 (64.2) | 182 (54.2) | **0.012** | 29 (10.6) | 38 (11.3) | 0.776 | 32 (11.7) | 73 (21.7) | **0.001** | |
| *Nectars* | 78 | 104 | 65 (83.3) | 87 (83.7) | 0.954 | 63 (80.8) | 83 (79.8) | 0.872 | 52 (66.7) | 60 (57.7) | 0.218 | 17 (21.8) | 19 (18.3) | 0.555 | 8 (10.3) | 27 (26.0) | **0.008** | |
| *Flavored drinks* | 69 | 58 | 64 (92.8) | 56 (96.6) | 0.350 | 47 (68.1) | 46 (79.3) | 0.156 | 61 (88.4) | 41 (70.7) | **0.012** | - | - | - | 5 (7.3) | 14 (24.1) | **0.008** | |
| *Carbonated drinks* | 57 | 83 | 40 (70.2) | 56 (67.5) | 0.735 | 20 (35.1) | 22 (26.5) | 0.276 | 22 (38.6) | 28 (33.7) | 0.555 | 5 (8.8) | 11 (13.3) | 0.413 | 6 (10.5) | 12 (14.5) | 0.495 | |
| *Dairy drinks* | 70 | 91 | 58 (82.9) | 83 (91.2) | 0.111 | 38 (54.3) | 55 (60.4) | 0.433 | 41 (58.6) | 53 (58.2) | 0.966 | 7 (10.0) | 8 (8.8) | 0.794 | 13 (18.6) | 20 (22.0) | 0.596 | |
| Foods | 609 | 699 | 255 (41.9) | 308 (44.1) | 0.425 | 159 (26.1) | 201 (28.8) | 0.285 | 191 (31.4) | 231 (33.1) | 0.516 | 17 (2.8) | 26 (3.7) | 0.348 | 13 (2.1) | 24 (3.4) | 0.158 | |
| *Bakery products* | 298 | 350 | 84 (28.2) | 103 (29.4) | 0.728 | 66 (22.2) | 83 (23.7) | 0.637 | 45 (15.1) | 62 (17.7) | 0.372 | 1 (0.3) | 0 (0.0) | 0.460 | 9 (3.0) | 14 (4.0) | 0.502 | |
| *Breakfast cereals* | 117 | 144 | 106 (90.6) | 123 (85.4) | 0.204 | 62 (53.0) | 74 (51.4) | 0.797 | 97 (82.9) | 114 (79.2) | 0.445 | 2 (1.7) | 3 (2.1) | 1.000 | 3 (2.6) | 5 (3.5) | 0.672 | |
| *Desserts* | 58 | 61 | 22 (37.9) | 29 (47.5) | 0.290 | 8 (13.8) | 9 (14.8) | 0.881 | 22 (37.9) | 25 (41.0) | 0.733 | 9 (15.5) | 15 (24.6) | 0.218 | 1 (1.7) | 4 (6.6) | 0.365 | |
| *Snacks* | 136 | 144 | 43 (31.6) | 53 (36.8) | 0.361 | 23 (16.9) | 35 (24.3) | 0.127 | 27 (19.9) | 30 (20.8) | 0.839 | 5 (3.7) | 8 (5.6) | 0.455 | 0 (0.0) | 1 (0.7) | 1.000 | |

Comparisons of proportions of marketing techniques, health and nutritional claims in products from pre- versus post-implementation period were made using Chi-squared and Fisher's exact tests. Bold values represent p<0.05

**Table S7. Marketing techniques, health and nutritional claims according to the front of package warning labels policy before and after front-of-package warning labels policy implementation, longitudinal sample**

|  | Post  Pre | Yes (n) | No (n) | P-value |
| --- | --- | --- | --- | --- |
| **Marketing techniques** |  |  |  |  |
| *Not high-in products in 2019 with at least 1 mkt technique (n=120)* | Yes (n) | 94 | 3 | NS |
|  | No (n) | 3 | 20 |  |
| Beverages (n=58) | Yes (n) | 44 | 1 | NS |
|  | No (n) | 0 | 13 |  |
| Foods (n=62) | Yes (n) | 50 | 2 | NS |
|  | No (n) | 3 | 7 |  |
| *High-in products in 2019 with at least 1 mkt technique (n=201)* | Yes (n) | 140 | 4 | **0.007** |
|  | No (n) | 17 | 40 |  |
| Beverages (n=37) | Yes (n) | 18 | 0 | NS |
|  | No (n) | 0 | 19 |  |
| Foods (n=164) | Yes (n) | 122 | 4 | **0.007** |
|  | No (n) | 17 | 21 |  |
| **Health Claims** |  |  |  |  |
| *Not high-in products in 2019 with at least 1 health claim (n=120)* | Yes (n) | 35 | 1 | NS |
|  | No (n) | 4 | 80 |  |
| Beverages (n=58) | Yes (n) | 19 | 0 | NS |
|  | No (n) | 0 | 39 |  |
| Foods (n=62) | Yes (n) | 16 | 1 | NS |
|  | No (n) | 4 | 41 |  |
| *High-in products in 2019 with at least 1 health claim (n=201)* | Yes (n) | 36 | 1 | **0.012** |
|  | No (n) | 10 | 154 |  |
| Beverages (n=37) | Yes (n) | 9 | 1 | NS |
|  | No (n) | 7 | 20 |  |
| Foods (n=164) | Yes (n) | 27 | 0 | NS |
|  | No (n) | 3 | 134 |  |
| **Nutritional Claims** |  |  |  |  |
| *Not high-in products in 2019 with at least 1 nutritional claim (n=120)* | Yes (n) | 96 | 2 | NS |
|  | No (n) | 1 | 21 |  |
| Beverages (n=58) | Yes (n) | 49 | 0 | NS |
|  | No (n) | 1 | 8 |  |
| Foods (n=62) | Yes (n) | 47 | 2 | NS |
|  | No (n) | 0 | 13 |  |
| *High-in products in 2019 with at least 1 nutritional claim (n=201)* | Yes (n) | 90 | 5 | NS |
|  | No (n) | 7 | 99 |  |
| Beverages (n=37) | Yes (n) | 33 | 0 | NS |
|  | No (n) | 0 | 4 |  |
| Foods (n=164) | Yes (n) | 57 | 5 | NS |
|  | No (n) | 7 | 95 |  |
| NS: not significant |  |  |  |  |
